# The Results of Pneumatic Balloon Dilatation Treatment in Children with Achalasia: A Single-Center Experience

**DOI:** 10.5152/tjg.2023.22328

**Published:** 2023-09-01

**Authors:** Selen Güler, Betül Aksoy, M. Onur Öztan, Yeliz Ç. Appak, Sinem Kahveci Çelik, Şenay Onbaşı Karabağ, Gökhan Köylüoğlu, Maşallah Baran

**Affiliations:** 1 Department of Pediatric Gastroenterology, Hepatology and Nutrition Clinic, Health Sciences University, Tepecik Training and Research Hospital, İzmir, Turkey; 2 Pediatric Surgery Clinic, Katip Celebi University & Health Sciences University Tepecik Training and Research Hospital, İzmir, Turkey; 3 Department of Pediatric Gastroenterology, Hepatology and Nutrition Clinic, Katip Celebi University & Health Sciences University, Tepecik Training and Research Hospital, İzmir, Turkey

**Keywords:** Achalasia, pneumatic balloon dilatation, children

## Abstract

**Background/Aims:**

Achalasia is a primary motility disorder characterized by a relaxation disorder of the lower esophageal sphincter. In pneumatic balloon dilatation, which is one of the treatment methods, the muscle fibers are torn with an endoscopically inflated balloon in the lower esophageal sphincter. This study aimed to evaluate the results of long-term pneumatic balloon dilatation treatment in our clinic for children diagnosed with achalasia.

**Materials and Methods:**

Pediatric patients who underwent pneumatic balloon dilatation with a diagnosis of achalasia in our pediatric gastroenterology clinic between 2016 and 2021 were included in the study. Demographic data of the patients, clinical findings at diagnosis, and follow-up results were evaluated retrospectively.

**Results:**

Ten patients who underwent 18 pneumatic balloon dilatation operations were included in the study. The mean follow-up period of the patients was 23.7 ± 14.1 months. It was observed that the procedure was performed once in 3 (30%) patients, twice in 2 (20%) patients, and 3 times in 3 (30%) patients. It is noteworthy that the diameter of the balloon used in the first procedure in patients who needed repeated operations was less than 30 mm. No complications were observed except for chest pain, which was detected in 1 patient.

**Conclusion:**

When the patients who needed recurrent dilatation were evaluated, it was noted that the diameter of the balloon in which the first procedure was performed in these patients was smaller. This study is an important contribution to the literature due to the scarcity of the pediatric achalasia data, in which long-term results related to pneumatic balloon dilatation are reported in Turkey.

**Main Points**

- The success rate of the pneumatic balloon dilatation (PBD) procedure in children with achalasia is 75%.
- The success rate is high in patients in whom a 30 mm balloon was used in the first PBD procedure.
- The complication rate of the PBD procedure is significantly low.
- Pneumatic balloon dilatation with a balloon with a diameter of 30 mm is an effective and safe initial treatment method for children with achalasia.

## Introduction

Achalasia is a primary motility disorder characterized by a relaxation disorder in the lower esophageal sphincter (LES). As the condition is rarer in children, the data on children are quite limited in our country, while more data in the literature are focused on adults. Pediatric achalasia is more common in boys between the ages of 7 and 15 years.^1,2^ Achalasia can be part of triple-A syndrome (achalasia, alacrimia, and adrenocortical insufficiency), and its association with Down syndrome has been reported.^3,4^ The most common symptoms are vomiting, dysphagia, and weight loss.^5^ The diagnosis is primarily based on clinical suspicion, but it can often be delayed due to the rarity of the disease. The Eckardt score was used to evaluate achalasia according to the symptoms. After evaluation with the Eckardt score, patients with a suspected diagnosis of achalasia were evaluated using a barium esophageal passage graph. A delay in the passage of barium into the stomach, observing a bird’s beak deformity around the lower esophagus, and the disappearance in the air pocket of the stomach can be evidence of achalasia. The gold standard method for the diagnosis and typing of achalasia is high-resolution manometry, but there are difficulties in its application in young children due to the problem of adaptation.^6^ Pharmacological treatment options, such as calcium channel blockers and endoscopic botulinum toxin, in the treatment of achalasia are rarely used in the pediatric population due to their short-term effectiveness and possible side effects.^7,8^ A laparoscopic Heller myotomy is the surgical treatment option used in achalasia. Peroral endoscopic myotomy (POEM) is a minimally invasive myotomy method in which the LES is reached by endoscopically creating a submucosal tunnel in the esophagus and dissecting muscle fibers, and the experience with this treatment in children is quite limited.^9^ In pneumatic balloon dilatation (PBD), muscle fibers are torn with a balloon that is endoscopically inflated in the LES. While the symptoms of pneumatic balloon dilatation decrease in the majority of patients at an early stage, the need for recurrent dilatation and surgery has been reported in the long term. However, there are not enough studies on long-term outcomes in children. This study aimed to evaluate the clinical findings and long-term results of PBD treatment in children diagnosed with achalasia who had undergone a PBD in our clinic.

## Materials and Methods

The data of pediatric patients who were diagnosed with achalasia and underwent pneumatic balloon dilatation between 2016 and 2021 at the Tepecik Education and Research Hospital Paediatric Gastroenterology Clinic were evaluated retrospectively. Patients whose diagnosis of achalasia was confirmed by esophageal manometry or barium passage graph after evaluation with the Eckardt score were included in the study. The demographic data of the patients, clinical findings and Eckardt scores in diagnosis and follow-up, imaging studies, methods of performing the PBD procedure, the presence of triple-A syndrome, the length of hospital stay, and postoperative follow-up results were evaluated. The informed consent form was obtained from the families of the patients.

### Eckardt Symptom Score Assessment

Dysphagia, regurgitation, retrosternal pain, and weight loss in kilograms are evaluated for the Eckardt symptom score, and each item is scored from 0 to 3. Cases with a score equal to and greater than 3 are considered active achalasia.^10^

### Pneumatic Balloon Dilatation Procedure

The endoscopic PBD procedure was performed on the patients under general anesthesia in the operating room during the day following night fasting. In the first PBD procedure, balloons of appropriate diameter (12-15-20-30 mm) were used according to the age and weight of the patients. The balloon dilator, which was advanced endoscopically via a guidewire, was placed at the lower end of the esophagus. Dilatation was performed by exposing the balloon inflated with air to pressure in the gastroesophageal junction under fluoroscopic control. In the balloon dilatation process, balloon diameter, balloon pressure, and dilatation process time were recorded. After dilatation, balloon air was drained, and the esophagus was pulled from its lower end. After the procedure, the lower end of the esophagus was checked with an endoscope for possible complications. After the procedure was terminated, the patients were placed on service monitoring. Cases without symptoms were given food 24 hours after the procedure. Patients who had no nutritional problems and did not develop complications were discharged.

The achievement of the PBD procedure was considered to be a decrease if the Eckardt score was below 3 on the postoperative days (day 1, day 15, day 30, 6 months, and 1 year). In the follow-up after discharge, the balloon diameter was increased, and the PBD procedure was performed again in patients with recurrent symptoms and an Eckardt score of 3 and higher in the cases. The treatment was considered successful in patients who were asymptomatic (Eckardt score <3) for at least 6 months after the last PBD procedure. The patients’ results over the first year and in the long term were evaluated.

An ethics committee approval was received from the ethical committee of the Tepecik Training and Research Hospital (2021/11-35).

### Statistical Analysis

Statistical analysis was performed using IBM’s Statistical Package for Social Sciences Statistics 24.0 package program (IBM Corp., Armonk, NY, USA). While categorical data are expressed as numbers and percentages (%), continuous data are expressed as mean ± SD.

## Results

The study included 10 patients. The mean follow-up period of the patients was 23.75 ± 14.1 months. The average age of diagnosis of the patients was 10.5 years (2-16 years), and 6 of them were female and 4 were male. The mean body weight *Z*-score at the time of application was determined to be –1.03 ± 0.83. Two of the patients were siblings, and 2 patients also had triple-A syndrome.

The median time for diagnosis was 10 months (1-84 months) after the patients’ complaints began. The mean Eckardt score of the patients at the time of diagnosis was 9.9 ± 1.5. When evaluating application complaints, 20% could not swallow solid food, 20% had difficulty swallowing, 20% felt stuck during feeding, 13.3% vomited undigested food, 13.3% wheezed and coughed, 6.6% rejected food, and among 6.6% weight loss was determined. Nine of the patients underwent esophageal passage x-rays before diagnosis and were reported to be compatible with achalasia. Endoscopic examination of the upper gastrointestinal tract was performed in 9 patients before PBD, and it was determined that all of them had dilatation in the proximal part of the esophagus and resistance in the transition to the distal part. Esophageal manometry was performed in 3 patients, 2 patients were evaluated as type II achalasia and 1 patient was evaluated as type I achalasia (Table 1).

We performed 18 PBDs. When the patients were reevaluated for the necessity of PBD procedures again, it was determined that 3 patients had been treated once, 2 patients had been treated twice, and 3 patients had been treated 3 times. The mean number of hospitalizations of the patients was 1.9 ± 0.87 times and the duration of hospitalization was 5.7 ± 4.05 days. The data of the patients in the first PBD and the results of a 1-year follow-up are shown in Table 1. The mean Eckardt scores of the patients after the first procedure were 1.20 ± 1.47 on day 1; 2.30 ± 4.27 on day 15, 1.37 ± 3.15 on day 30, 2.43 ± 3.82 at 6 months, and 3.16 ± 4.66 at 1 year. The balloon diameter of the patients used in the first procedure and the Eckardt scores after the procedure are shown in Table 1.

The median Eckardt scores of the patients on whom a 30 mm diameter balloon was used in the first procedure were recorded as follows: 0.5 (0-3) on day 1; 0 (0-0) on day 15, 0 (0-0) on day 30, 0 (0-1) at 6 months, and 1.5 (0-4) at 1 year. The median Eckardt scores of patients on whom balloons with a diameter of less than 30 mm were used were as follows: 1 (0-4) on day 1, 1.5 (0-12) on day 15, 1 (0-9) on day 30, 8 (0-8) at 6 months, and 6 (0-12) at 1 year. The Eckardt scores of the patients after the PBD procedure performed on day 1 and day 15 when a 30 mm diameter balloon and when balloons with a diameter of less than 30 mm were used were compared. There was no statistically significant difference between the days (*P* = .91 and *P* = .25, respectively).

Patients 1, 2, and 3 were followed up without clinical signs after a once-performed PBD procedure. Patient 6 had a POEM operation in the first month after the PBD procedure due to family preferences.

When the complications of the PBD procedure in patients were evaluated, severe chest pain, pneumonia, and pleural effusion had developed in patient 1 after the first procedure. No complications were observed in the other patients.

The detected success rates were as follows: day 1, 80%; day 15, 70%; day 30, 87.5%; 6 months, 71.4%; and 1 year, 50%. Patients 4, 5, 7, 8, 9, and 10 underwent a second PBD. The median duration of the second procedure was 8.75 months. The data for these patients are shown in Table 2. The mean Eckardt scores of the patients after the second balloon dilatation procedure were 0.33 ± 0.81 on day 1, 0.33 ± 0.81 on day 15, 1.67 ± 2.33 on day 30, 4.5 ± 3.00 at 6 months, and 6.66 ± 6.11 at 1 year. The success rates were 100% on day 1, 100% on day 15, and 83% on day 30. No complications were observed in the patients after the second procedure. The third balloon dilatation was performed on patients 8, 9, and 10. The median duration of the second procedure was 16 months. The data for these patients are shown in Table 3.

No complications were observed in the patients after the third procedure. The mean Eckardt scores of the patients recorded after the third balloon dilatation procedure were on day 1, on day 15, on day 30, and at 6 months 0.33 ± 0.57 and 1.0 ± 1.73 at 1 year. The success rates were determined as 100% on day 1, day 15, and day 30 and after 6 months and 1 year. Patient 8 underwent a surgical myotomy operation at the 14th month after the third procedure due to the fact that the Eckardt score was 3.

The mean duration of clinical well-being of the patients who underwent only the PBD treatment after the last procedure was 19.12 (4-38) months, and the treatment success rate of these 8 patients was 75% (Table 4).

## Discussion

In our study, a success rate of 75% was found only in patients who underwent a PBD procedure. The success rate was high in our achalasia patients in whom a 30 mm balloon was used in the first PBD procedure. In addition, a second PBD was required for patients in whom balloons with a diameter of less than 30 mm were used, and treatment success could only be achieved in one of these patients. A third PBD procedure was performed on other patients, using a 30 mm balloon; in these patients, treatment success was achieved at the 1-year follow-up, except for the patient who underwent a myotomy. In a study conducted on adult patients, the balloon diameter was noted, and as we found in our study, a PBD with a balloon with a diameter of 30 mm was found to be an effective and safe initial treatment method for achalasia.^11^

In our study, it was also necessary to repeat the PBD in children at ≤8 years of age. In a systematic review conducted on pediatric patients, the failure rate was found to be higher in patients treated with a PBD under the age of 10, in a similar way as in our study.^12^ In a review evaluating treatment options in childhood achalasia, PBD was recommended for children older than 8 years.^13^ In another study where the results of PBD treatment of pediatric achalasia patients were examined, it was found that the only negative predictive factor was being under the age of 6. In the same study, the success rate of balloon dilatation was found to be 87%.^14^ In our study, when patients with recurrent dilatation needs were evaluated, it was observed that the balloon diameter of the first procedure was lower for these patients. It was observed that the symptoms of the patients decreased with increasing balloon diameters in recurrent dilatations. In a study conducted by Boeckxstaens et al^15^ with 201 adult patients, it was found that the effect of pneumatic dilatation and Heller myotomy on Eckardt scoring was equal at the 2-year follow-up. The same cohort was reported again after 5 years, and 25% in the dilatation group needed a re-dilatation, but there was no significant difference in the success rate between the 2 treatments.^16^ Pastor et al^17^ evaluated patients who had undergone PBDs and reported that 17% of the patients did not need further intervention, while 53% said that they needed more dilatation. However, the patients did not need surgical intervention in the long term. In our study, 30% of the patients did not need additional intervention, 60% underwent a recurrent dilatation procedure, a patient underwent a myotomy after the third balloon dilatation procedure, and 1 patient underwent the first POEM operation after the PBD, due to the family’s own request.

There are various discussions on the treatment models of achalasia in childhood, and there is no definite consensus. A total of 108 achalasia children were evaluated in a study involving 38 centers from 24 countries, and it was found that 58% preferred Heller myotomy for treatment, followed by 46% PBD and 29% POEM for treatment.^18^ A PBD is the most powerful alternative to surgical treatment methods due to the fact that the complication rates are lower. Peroral endoscopic myotomy is increasingly being used in pediatric settings with promising short-term results. When comparing POEM and PBD in children and adults, the American Society of Gastrointestinal and Endoscopic Surgeons has recommended POEM as a priority, but for patients concerned about the long-term use of proton pump inhibitors, it has been recommended that the patient and surgeon decide together between these 2 methods. In comparison with Heller myotomy and POEM in adult and pediatric patients with type I and type II achalasia, the patient and the surgeon decide together to perform POEM or Heller myotomy. In patients with type III achalasia, POEM is recommended to be performed first.^19^ A study conducted by Yadlapati and Gupta^20^ found that the 92% success rate of POEM was significantly higher compared to the 54% success rate of PBD; however, gastroesophageal reflux disease (GERD)-associated reflux esophagitis was significantly higher in the POEM arm (41%) than in the PBD arm (7%). Gastroesophageal reflux disease may occur after surgery in cases of achalasia due to disruption of the LES; therefore, careful follow-up is required, especially in children treated with POEM.^21^ A recent systematic review, analyzing over 1122 patients from 22 studies, reported subcutaneous emphysema in 32%, capno/pneumothorax in 11%, and capno/pneumoperitoneum in 31% of cases.^22^ Serious complications were reported to occur at a rate of 0.3% for mediastinal leaks, 1.1% for postoperative bleeding, and 0.09% for mortality perforation, with pneumothorax, subcutaneous emphysema, and capnoperitoneum being the main reported complications of the POEM process.^23^ Compared to these complications, the complication rate in our study was significantly lower.

The most commonly reported symptoms in achalasia are vomiting (80%), dysphagia (75%), weight loss (64%), respiratory symptoms (44%), chest pain/painful swallowing (45%), growth retardation (31%), and nocturnal regurgitation (21%).^5^ Regurgitation of undigested foods, cough, aspiration, and pneumonia may also occur.^19^ In our study, swallowing was observed to be the most common problem in our patients. In most pediatric patients with achalasia symptoms, primary GERD can be considered and the diagnosis of achalasia may be delayed by trying long-term reflux treatment. It has been reported that the diagnosis may be delayed by 6-10 years in cases of achalasia.^24^ In our study, it was observed that the patients were diagnosed in the median 10th month after the onset of symptoms. Clinical findings should be questioned in more detail in patients who are considered to be diagnosed with reflux but cannot be responded to, and achalasia should be considered in a separate diagnosis. The limitations of our study were that our study was retrospective, the number of patients was low, and our follow-up period was not very long.

As a result, our study is important for its contribution to the literature because there are few data on children’s achalasia in which long-term results for PBD have been reported in our country. Although achalasia is rare in childhood, increasing awareness of this issue will ensure that the diagnosis is made at an earlier age in pediatric patients and that appropriate treatment is applied. For patients in childhood, PBD is an important treatment option in terms of being a less invasive intervention and receiving a treatment response due to the inability to get an adequate response with medical treatments in achalasia and its side effects. Since the Eckardt score, in which symptom questioning is performed, questions the decrease in body weight in kilograms, its use is limited in pediatric patients. Therefore, in terms of early recognition of achalasia in pediatric patients, a pediatric adjustment of the Eckardt score is needed. There is also a need for studies in which the use of PBD in childhood is evaluated and a larger number of cases are monitored over a longer period of time.

## Figures and Tables

**Table 1.** Patient Data on the First Pneumatic Balloon Dilatation and 1-Year Follow-Up Findings

n	Gender	Age	BW *Z*-Score	Eckart Score in Diagnosis	Type of Achalasia	Balloon Diameter (mm)	Balloon Pressure (atm)	Duration of the Operation (min)	Eckart Score, Day 1	Eckardt Score, Day 15	Eckardt Score, Day 30	Eckardt Score 6, Months	Eckardt Score, 1 Year
1	Male	16	0.90	9		20	3	3	4	0	0	0	0
2	Male	15	–1.72	8	Type II	30	2	2	0	0	0	0	0
3	Male	16	–0.88	10	Type II	30	1.5	2	1	0	0	0	0
4	Male	14	–0.73	10	Type II	30	2	2	3	0	0	1	4
5	Female	6	–1.24	11		30	2	2	0	0	0	0	3
6	Female	8	–1.11	12		20	2	2	2	12			
7	Female	6	–2.10	10		12	2	2	0	8	9		
8	Female	6	–1.66	7		20	2	2	0	0	0	8	
9	Female	2	–.52	12		11	2	2	0	0	2	8	12
10	Female	16	–1.24	10		11	2	2	2	3			

BW, body weight.

**Table 2.** Data of the Patients Who Underwent the Second Pneumonic Balloon Dilatation Procedure and the Results of a 1-Year Follow-up

n	Period Between First and Second PBD	Balloon Diameter (mm)	Balloon Pressure (atm)	Duration of the Operation (dk)	Eckardt Score, Day 1	Eckardt Score, Day 15	Eckardt Score, 1 Month	Eckardt Score, 6 Months	Eckardt Score, 1 Year
4	12 months	30	2	2	0	0	0	—	—
5	12 months	30	2	2	0	0	0	—	—
7	1 month	15	2	2	0	0	0		
8	6 months	15	2	2	0	0	6	6	
9	11.5 months	12	2	2	0	0	2	6	12
10	15 days	20	2	2	2	2	2	6	8

PBD, pneumatic balloon dilatation.

**Table 3.** Data of the Patients who Underwent the Third Pneumatic Balloon Dilatation Procedure and the Results of a 1-Year Follow-up

n	Period Between Second and Third PBD	Balloon Diameter (mm)	Balloon Pressure (atm)	Duration of the Operation (dk)	Eckardt Score, Day 1	Eckardt Score, Day 15	Eckardt Score, 1 Month	Eckardt Score, 6 Months	Eckardt Score, 1 Year
8	6 months	30	2	2	1	1	1	1	2
9	16 months	12	2	2	0	0	0	0	1
10	22 months	30	2	2	0	0	0	0	0

PBD, pneumatic balloon dilatation.

**Table 4.** Number of PBD Procedures and Follow-up Periods After the Last Procedure and the Last Eckardt Scores of the Patients

n	Number of PBD	Follow-up Period After the Last Procedure (month)	Eckardt Score
1	1	12	0
2	1	15	0
3	1	25	0
4	2	4	0
5	2	4	0
7	2	38	0
9	3	26	1
10	3	29	1

PBD, pneumatic balloon dilatation.
